# Supplementary material for: ECNano: A cost-effective workflow for target enrichment sequencing and accurate variant calling on 4800 clinically significant genes using a single MinION flowcell
Source: BMC Med Genomics. 2022 Mar 4;15:43. doi: 10.1186/s12920-022-01190-3 (PMC8895767; doi:10.1186/s12920-022-01190-3)
Supplement: Supplementary file 2 — Additional file 2: A step-by-step wet-lab protocol of ECNano library preparation including the detailed procedures in (1) input DNA preparation, (2) DNA fragmentation and size selection, (3) DNA repair and end-prep, (4) amplification and target capture, and (5) ONT library preparation. [file 12920_2022_1190_MOESM2_ESM.docx]

**Protocol for Targeted Medical Exome Sequencing Capture (SQK-LSK109) (Document ID: 00008):**

***Optimized from Nanopore Protocol Sequence capture (SQK-LSK109) Version: SCE_9075_v109_revM_14Aug2019 for medical exome target capture**

1. **Preparing input DNA:**

Materials: 3.5 µg high molecular weight genomic DNA

Consumables: 1.5 mL Eppendorf DNA LoBind tubes, 10 mM Tris-HCL pH 8.0 buffer

Equipment: Microfuge, Nanodrop, Qubit Fluorometer

1. Measure purity of sample with Nanodrop using 1 µL of sample.

| **gDNA** | µg/µL | 260/230 (1.9-2.2) | 260/280 (1.7-2) | Pass or fail |
| --- | --- | --- | --- | --- |
| sample |  |  |  |  |

1. Quantify 1µl of sample using Qubit fluorometer.

| **gDNA** | µg/uL | vol (µl) for 3.5 µg of gDNA | vol (µL) of Tris-HCL Buffer |
| --- | --- | --- | --- |
| sample |  |  |  |

1. Transfer 3.5 μg genomic DNA into a clean 1.5 mL Eppendorf DNA LoBind tube.
2. Adjust the volume to 130 μL with Tris-HCL(pH 8.0) buffer.
3. Mix thoroughly and spin down briefly in a microfuge.
4. **Fragment DNA and Size selection:**

Materials: 3.5 µg genomic DNA in 130 μL Tris-HCL

Consumables: Covaris microTUBE AFA Fiber Pre-Slit Snap-Cap (520045), 1.5 mL Eppendorf DNA LoBind tubes, AMPure XP beads, Absolute ethanol, Nuclease-free water, 10 mM Tris-HCl pH 8.0 buffer, Milli-Q water

Equipment: M220 Focused-ultrasonicator, Nanodrop, Qubit Fluorometer, Vortex, Microfuge, Magnetic Rack (1.5 mL)

1. Transfer each sample of 3.5 µg genomic DNA in 130 μL Tris-HCL buffer to a Covaris microTUBE.
2. Fill the acoustic assembly of the ultrasonicator with 15 mL Milli-Q water.
3. Place the microTUBE into the aperture at the center of the tube holder of the acoustic assembly and make sure the tube sits all the way down in the holder.
4. Close the safety cover and set the method to target ~1500 bp peak as shown below:

| Peak Incident Power (W) | Duty Factor (%) | Cycles per Burst (cpb) | Treatment Time (sec) | Temperature  (°C) |
| --- | --- | --- | --- | --- |
| 50 | 2 | 200 | 20 | 20 |

1. Remove microTUBE and transfer 130 μL fragmented DNA to a clean 1.5 mL Eppendorf DNA LoBind tube.
2. Prepare AMPure XP beads at room temperature for use; resuspend by vortexing .
3. Add 0.6x of resuspended AMPure XP beads to 130 μL fragmented DNA and mix gently by pipetting.
4. Incubate for 10 minutes at RT, agitating every few minutes.
5. Prepare 500 μL of fresh 80% ethanol in nuclease-free water.
6. Spin down the sample and pellet on a magnet until the supernatant is clear and colourless. Keep the tube on the magnet, and pipette off the supernatant into a clean 1.5 mL Eppendorf DNA LoBind tube (labeled as supernatant).
7. Keep on magnet, wash beads with 200 µL of freshly prepared 80% ethanol without disturbing the pellet.
8. Remove the 80% ethanol using a pipette and discard.
9. Repeat steps 2.11-2.12.
10. Spin down and place the tube back on the magnet. Pipette off any residual ethanol.
11. Allow to dry for ~2 minutes, but do not dry the pellet to the point of cracking.
12. Remove the tube from the magnetic rack and resuspend the pellet in 50 µL of Tris-HCl buffer.
13. Gently agitate the tube to ensure that beads are covered in the buffer and incubate for 10 minutes at RT, gently agitating the tube every few minutes to aid resuspension of the pellet.
14. Pellet the beads on a magnet until the eluate is clear and colourless.
15. Extract 49 µL of eluate into a clean 1.5 mL Eppendorf DNA LoBind tube.
16. Analyse 1µL DNA from 2.19 and supernatant from 2.10 for:

1. Nanodrop:

| **Size-Selected DNA** | µg/µL | 260/230 (1.9-2.2) | 260/280 (1.7-2) | Pass or fail |
| --- | --- | --- | --- | --- |
| sample |  |  |  |  |
| sample supernatant |  |  |  |  |

2. Qubit:

| **Size-Selected DNA** | µg/µL | total quantity (µg) |
| --- | --- | --- |
| sample |  |  |
| sample supernatant |  |  |

3. Agarose gel for fragment size (optional):

Ladder: Invitrogen 100 bp DNA Ladder or any other that range of 100 bp to 2,000 bp.

1% Agarose gel, pre-stain, 100V, 20mins.

1. **DNA repair and End-prep:**

Materials: Size-selected DNA in 48 µL

Consumables: NEBNext FFPE Repair Mix (M6630), NEBNext End Repair/ dA-tailing Module (E7546), 1.5 mL Eppendorf DNA LoBind tubes, 0.2 mL thin-walled PCR tubes, AMPure XP beads, Absolute ethanol, Nuclease-free water, 10 mM Tris-HCl pH 8.0 buffer

Equipment: Thermal cycler, Microfuge, Vortex, Nanodrop, Qubit Fluorometer, Magnetic Rack (1.5 mL)

1. Prepare reagent mastermix in a 0.2 mL thin-walled PCR tube for FFPE DNA repair treatment and End-repair/ dA-tailing module:

| Reagent | Volume |
| --- | --- |
| Size-selected genomic DNA | 48 µL |
| FFPE Repair Buffer | 3.5 µL |
| FFPE Repair Mix | 2 µL |
| Ultra II End-prep reaction buffer | 3.5 µL |
| Ultra II End-prep enzyme mix | 3 µL |
| Total | **60 µL** |

1. Mix gently by flicking the tube, and spin down.
2. Incubate the reaction for 15 minutes at 20° C and 15 minutes at 65° C in a thermal cycler.
3. Prepare AMPure XP beads for use; resuspend by vortexing.
4. Transfer the sample to a 1.5 ml DNA LoBind Eppendorf tube.
5. Add 0.6x of resuspended AMPure XP beads to FFPE repair reaction and mix gently by pipetting.
6. Incubate for 10 minutes at RT, agitating every few minutes.
7. Prepare 500 μL of fresh 80% ethanol in nuclease-free water.
8. Spin down the sample and pellet on a magnet until the supernatant is clear and colourless. Keep the tube on the magnet, and pipette off the supernatant into a clean 1.5 mL Eppendorf DNA LoBind tube (labeled as supernatant).
9. Keep on magnet, wash beads with 200 µL of freshly prepared 80% ethanol without disturbing the pellet.
10. Remove the 80% ethanol using a pipette and discard.
11. Repeat steps 3.10-3.11.
12. Spin down and place the tube back on the magnet. Pipette off any residual ethanol.
13. Allow to dry for ~2 minutes, but do not dry the pellet to the point of cracking.
14. Remove the tube from the magnetic rack and resuspend the pellet in 32 µL of Tris-HCl buffer.
15. Gently agitate the tube to ensure that beads are covered in the buffer and incubate for 10 minutes at RT, gently agitating the tube every few minutes to aid resuspension of the pellet.
16. Pellet the beads on a magnet until the eluate is clear and colourless.
17. Extract 31 µL of eluate into a clean 1.5 mL Eppendorf DNA LoBind tube.
18. Analyse 1 µL of the FFPE-repaired DNA from 3.18 and supernatant from 3.9 for:

Qubit:

| **FFPE-EP DNA** | µg/µL | total quantity (µg) |
| --- | --- | --- |
| sample |  |  |
| sample supernatant |  |  |

1. Take forward 700ng FFPE-repaired DNA into adaptor ligation.
2. **Ligation of PCR adapters:**

Materials: Size-selected DNA in 30 µL, PCR adapter (PCA) from EXP-PCA001 kit

Consumables: Nuclease-free water, NEB Blunt/TA Ligase Master Mix (M0367), Agencourt AMPure XP beads, Absolute ethanol, 1.5 mL Eppendorf DNA LoBind tubes, 10 mM Tris-HCl pH 8.0 buffer

Equipment: Microfuge, Vortex, Qubit Fluorometer, Magnetic Rack (1.5 mL)

1. Add the reagents in the order given below, mixing by flicking the tube between each sequential addition:

| Reagent | Volume |
| --- | --- |
| 700ng End-prepared DNA | 30 µL |
| PCR Adapters (PCA) | 20 µL |
| NEB Blunt/TA Ligase Master Mix | 50 µL |
| Total | **100 µL** |

1. Mix gently by flicking the tube, and spin down.
2. Incubate the reaction for 10 minutes at room temperature.
3. Prepare the AMPure XP beads for use; resuspend by vortexing.
4. Add 0.6x of resuspended AMPure XP beads to the reaction and mix by pipetting.
5. Incubate for 10 minutes at RT, agitating every few minutes.
6. Prepare 500 μL of fresh 80% ethanol in nuclease-free water.
7. Spin down the sample and pellet on a magnet until the supernatant is clear and colourless. Keep the tube on the magnet, and pipette off the supernatant into a clean 1.5 mL Eppendorf DNA LoBind tube (labeled as supernatant).
8. Keep on magnet, wash beads with 200 µL of freshly prepared 80% ethanol without disturbing the pellet.
9. Remove the 80% ethanol using a pipette and discard.
10. Repeat steps 4.9-4.10.
11. Spin down and place the tube back on the magnet. Pipette off any residual ethanol.
12. Allow to dry for ~2 minutes, but do not dry the pellet to the point of cracking.
13. Remove the tube from the magnetic rack and resuspend the pellet in 50 µL of Tris-HCl buffer.
14. Gently agitate the tube to ensure that beads are covered in the buffer and incubate for 10 minutes at RT, gently agitating the tube every few minutes to aid resuspension of the pellet.
15. Pellet the beads on a magnet until the eluate is clear and colourless.
16. Extract 49 µL of eluate into a clean 1.5 mL Eppendorf DNA LoBind tube.
17. Analyse 1 µL of the ligated DNA from 4.17 and supernatant from 4.8 for:

Qubit:

| **Ligated DNA** | µg/µL | total quantity (µg) |
| --- | --- | --- |
| sample |  |  |
| sample supernatant |  |  |

1. **PCR:**

Materials: Primer Mix (PRM) from EXP-PCA001 kit

Consumables: 2x Long AMP Taq (M0287), Agencourt AMPure XP beads, Nuclease-free water, Absolute ethanol, 1.5 mL Eppendorf DNA LoBind tubes, 0.2 mL thin-walled PCR tubes, 10 mM Tris-HCl pH 8.0 buffer

Equipment: Thermal cycler, Ice bucket with ice, Magnetic Rack (1.5 mL), Vortex, Qubit Fluorometer, Microfuge

1. In a 0.2 mL thin-walled PCR tube mix the following:

| Reagent | Volume |
| --- | --- |
| 2x Long Amp Taq | 50 µL |
| PRM Adapters (10 μM) | 2 µL |
| Template DNA | 48 µL |
| Total | **100 µL** |

1. Amplify using the following cycling conditions:

| **Cycles step** | **Temperature** | **Time** | **No. of cycles** |
| --- | --- | --- | --- |
| Initial denaturation | 95°C | 3 mins | 1 |
| Denaturation | 98°C | 20 secs | 14 |
| Annealing | 62°C | 15 secs | 14 |
| Extension | 65°C | 3 mins | 14 |
| Final extension | 65°C | 3 mins | 1 |
| Hold | 4°C | ∞ |  |

1. Prepare the AMPure XP beads for use; resuspend by vortexing.
2. Transfer the sample to a 1.5 mL DNA LoBind Eppendorf tube.
3. Add 0.6x of resuspended AMPure XP beads to the reaction and mix by pipetting.
4. Incubate for 10 minutes at RT, agitating every few minutes.
5. Prepare 500 μL of fresh 80% ethanol in nuclease-free water.
6. Spin down the sample and pellet on a magnet until the supernatant is clear and colourless. Keep the tube on the magnet, and pipette off the supernatant into a clean 1.5 mL Eppendorf DNA LoBind tube (labeled as supernatant).
7. Keep on magnet, wash beads with 200 µL of freshly prepared 80% ethanol without disturbing the pellet.
8. Remove the 80% ethanol using a pipette and discard.
9. Repeat steps 5.9-5.10.
10. Spin down and place the tube back on the magnet. Pipette off any residual ethanol.
11. Allow to dry for ~2 minutes, but do not dry the pellet to the point of cracking.
12. Remove the tube from the magnetic rack and resuspend the pellet in 36 µL of Tris-HCl buffer.
13. Gently agitate the tube to ensure that beads are covered in the buffer and incubate for 10 minutes at RT, gently agitating the tube every few minutes to aid resuspension of the pellet.
14. Pellet the beads on a magnet until the eluate is clear and colourless.
15. Extract 35 µL of eluate into a clean 1.5 mL Eppendorf DNA LoBind tube.
16. Analyse 1 µL of the amplified DNA from 5.17 and supernatant from 5.8 for:

1. Qubit:

| **Amplified DNA** | µg/µL | total quantity (µg) |
| --- | --- | --- |
| sample |  |  |
| sample supernatant |  |  |

The resultant amplicon should be around 1 µg to 2 µg.

2. Agarose gel for fragment size (optional):

Ladder: Invitrogen 100 bp DNA Ladder or any other that range of 100 bp to 2,000 bp.

1% Agarose gel, pre-stain, 100V, 20mins.

1. **Hybridisation:**

Materials: SureSelect TE Reagent Kits (G9605A), Agilent Sureselect XT Focused Exome (5190-7787)

Consumables: Cot-1 DNA (ThermoFisher Scientific 15279- 011), Nuclease-free water, 0.2 mL thin-walled PCR tubes, Blocking oligo at 1 mM, sequence 5'-AGGTTAAACACCCAAGCAGACGCCGCAATATCAGCACCAACAGAAACAA 3'

Equipment: SpeedVac, Thermal cycler, Ice bucket with ice, Vortex, Microfuge

1. In a clean 1.5 mL Eppendorf DNA LoBind tube, mix the following:

| Reagent | Volume |
| --- | --- |
| DNA library | 1000 ng |
| Cot-1 DNA | 5 µg |
| Blocking oligo top | 1 µL |

The volume of the reaction can be variable, as the water is evaporated in step 2. After this, the DNA is reconstituted to a consistent volume across all samples.

1. Evaporate the water in a SpeedVac at medium temperature (45°C) for at least 30 mins.
   1. For each sample library prepared, do one hybridisation and capture.
   2. Break off the cap, cover with parafilm, and poke holes on it with a narrow gauge needle.
2. Reconstitute with nuclease-free water to a final volume of 9 μL. Pipette up and down along the sides of the tube for optimal recovery.
3. Mix thoroughly by vortexing and spin down for 1 minute.
4. Move the 9 μL gDNA library samples to 0.2 mL thin-walled PCR tubes. Incubate the tube in the thermal cycler using the following program:

| **Stage** | **Temperature** | **Time** |
| --- | --- | --- |
| Step 1 | 95°C | 5 mins |
| Step 2 | 65°C | 5 mins |
| Step 3 | 65°C | Hold |

1. Prepare the Hybridisation Buffer mix and the RNase Block to be combined with the Capture Library reagent from the SureSelect kit. This will then be combined with the adapted amplified DNA sample.
2. Once the sample tubes are in the thermal cycler, mix the reagents in the table below to make the Hybridisation Buffer:

| Reagent | Volume for 1 reaction |
| --- | --- |
| SureSelect Hyb 1 (orange cap or bottle) | 6.63 µL |
| SureSelect Hyb 2 (red cap) | 0.27 µL |
| SureSelect Hyb 3 (yellow cap or bottle) | 2.65 µL |
| SureSelect Hyb 4 (black cap or bottle) | 3.45 µL |
| Total | **13 µL** |

In the event of precipitation, warm the Hybridisation Buffer at 65 °C for 5 minutes. Otherwise, keep the buffer at room temperature until it is used for the Hybridisation mix.

1. Dilute the SureSelect RNase Block (purple cap) in nuclease-free water, sufficient for the number of hybridisation reactions in the run. Keep the mixture on ice.

| RNase Block dilution  (parts RNase Block:water) | Volume of dilute RNase Block  Required per hybridization reaction |
| --- | --- |
| 25% (1:3) | 2 µL |

1. Prepare the Capture Library Hybridisation Mix according to the table below. Only keep the mixture at room temperature until it is added to sample.

| Reagent | Volume for 1 reaction |
| --- | --- |
| Hybridisation buffer mixture | 13 µL |
| 25% RNase Block solution | 2 µL |
| XT Focused Exome | 5 µL |
| Total | **20 µL** |

1. Keeping all reagents at 65 °C, add 20 μL of the Capture Hybridisation Mix to each tube containing 9 μL adapted and amplified DNA sample.
2. Mix by pipetting.
3. Seal all the caps on the tubes.
4. Incubate the tubes for 16 to 24 hours at 65 °C with a heated lid set at 105 °C.
5. **Pull-down:**

Materials: SureSelect TE Reagent Kits (G9605A)

Consumables: Dynabeads MyOne Streptavidin T1 (ThermoFisher Scientific, 65601), Nuclease-free water, 0.2 mL thin-walled PCR tubes, 1.5 mL Eppendorf DNA LoBind tube

Equipment: Thermal cycler, Vortex, Magnetic Rack (0.2 mL), Plate mixer, Microfuge, Ice bucket with ice

**Important:** It is important to maintain bead suspensions at 65 °C during the washing procedure below to ensure specificity of capture. **Make sure that the SureSelect Wash Buffer 2 is pre-warmed to 65 °C before use**. Do not use a tissue incubator, or other devices with significant temperature fluctuations, for the incubation steps.

1. Warm the SureSelect Wash Buffer 2 at 65 °C.
2. Resuspend the Dynabeads MyOne Streptavidin T1 magnetic beads by vortexing at room temperature.
3. Add 50 μL of the beads to wells of a fresh 0.2 mL thin-walled PCR tube, one tube for each hybridisation sample.
4. Add 200 μL of SureSelect Binding Buffer to the beads.
5. Mix by pipetting.
6. Place on a magnetic rack, allow beads to pellet and pipette off supernatant.
7. Repeat steps 7.4 - 7.6 twice more for a total of three washes.
8. Resuspend the beads in 200 μL of SureSelect Binding Buffer.
9. Keep the tube at 65 °C. Transfer the whole volume (approximately 25 to 29 μL) of each hybridisation mixture from the 65 °C reaction to the tube containing 200 μL of washed streptavidin beads.
10. Pipette up and down until beads are fully resuspended.
11. Seal all the caps on the tubes.
12. Incubate the tube on a 96-well plate mixer, mixing at 1400 rpm for 30 minutes at room temperature. Make sure the samples are firmly fixed on the mixer plate if single tubes were used.
13. Spin down the sample and pellet on a magnet until the supernatant is clear and colourless. Keep the tube on the magnet, and pipette off the supernatant into a clean 1.5 mL Eppendorf DNA LoBind tube (labeled as supernatant).
14. Resuspend the beads in 200 μL of SureSelect Wash Buffer 1.
15. Pipette up and down until beads are fully resuspended.
16. Incubate the reaction for 15 minutes at room temperature.
17. Spin down the sample and pellet on a magnet until the supernatant is clear and colourless. Keep the tube on the magnet, and pipette off the supernatant.
18. Resuspend the beads in 200 μL of Wash Buffer 2 pre-warmed at 65 °C.
19. Pipette up and down until beads are fully resuspended.
20. Seal all the caps on the tubes.
21. Incubate the tube for 10 minutes at 65 °C on the thermal cycler.
22. Pellet the sample on a magnet until the supernatant is clear and colourless. Keep the tube on the magnet, and pipette off the supernatant.
23. Repeat the wash steps 7.18 - 7.22 twice more for a total of three washes. Make sure all of the wash buffer has been removed during the final wash.
24. Add 96 μl of nuclease-free water to each sample, and pipette up and down to resuspend the beads. Keep the samples on ice.
25. Captured DNA remains on the streptavidin beads during the post-capture amplification step.
26. **Elution and amplification of DNA:**

Materials: Primer Mix (PRM) from EXP-PCA001 kit

Consumables: 0.2 mL thin-walled PCR tubes, 2x Long AMP Taq, Nuclease-free water, Agencourt AMPure XP beads, 10 mM Tris-HCl pH 8.0 buffer, Absolute ethanol

Equipment: Thermal cycler, Ice bucket with ice, Vortex, Magnetic Rack (0.2 mL), Magnetic Rack (1.5 mL)

1. Split the sample into two 48 μL aliquots.
2. Prepare the following reaction in duplicate. In 0.2 mL thin-walled PCR tubes mix the following:

| Reagent | Volume |
| --- | --- |
| 2x Long Amp Taq | 50 μL |
| PRM Adapters (10 μM) | 2 μL |
| Template DNA | 48 μL |
| Total | **100 μL** |

1. Amplify using the following cycling conditions:

| **Cycles step** | **Temperature** | **Time** | **No. of cycles** |
| --- | --- | --- | --- |
| Initial denaturation | 95°C | 3 mins | 1 |
| Denaturation | 98°C | 20 secs | 17 |
| Annealing | 62°C | 15 secs | 17 |
| Extension | 65°C | 3 mins | 17 |
| Final extension | 65°C | 3 mins | 1 |
| Hold | 4°C | ∞ |  |

1. Place the amplified sample on a magnetic rack. Once the solution is clear, transfer the supernatant into a clean 1.5 mL DNA Lo-Bind Eppendorf tube. The beads can now be discarded.
2. Prepare the AMPure XP beads for use; resuspend by vortexing.
3. Add 0.6x of resuspended AMPure XP beads to the reaction and mix by pipetting.
4. Incubate for 10 minutes at RT, agitating every few minutes.
5. Prepare 500 μL of fresh 80% ethanol in nuclease-free water.
6. Spin down the sample and pellet on a magnet until the supernatant is clear and colourless. Keep the tube on the magnet, and pipette off the supernatant into a clean 1.5 mL Eppendorf DNA LoBind tube (labeled as supernatant).
7. Keep on magnet, wash beads with 200 µL of freshly prepared 80% ethanol without disturbing the pellet.
8. Remove the 80% ethanol using a pipette and discard.
9. Repeat steps 8.10-8.11.
10. Spin down and place the tube back on the magnet. Pipette off any residual ethanol.
11. Allow to dry for ~2 minutes, but do not dry the pellet to the point of cracking.
12. Remove the tube from the magnetic rack and resuspend the pellet in 27 µL of Tris-HCl buffer.
13. Gently agitate the tube to ensure that beads are covered in the buffer and incubate for 10 minutes at RT, gently agitating the tube every few minutes to aid resuspension of the pellet.
14. Pellet the beads on a magnet until the eluate is clear and colourless.
15. Extract 26 µL of eluate into a clean 1.5 mL Eppendorf DNA LoBind tube. Pool the two samples together to yield a 52 μL eluted sample.
16. Analyse 1 µL of the amplified DNA from 4.18 and supernatant from 4.9 for:

1. Qubit:

| **Amplified DNA** | µg/μL | total quantity (µg) |
| --- | --- | --- |
| sample |  |  |
| sample supernatant |  |  |

The resultant amplicon should be > 1 µg. If the yield is under 700 ng, this indicates failure of the target capture.

2. Agarose gel for fragment size (optional):

Ladder: Invitrogen 100 bp DNA Ladder or any other that range of 100 bp to 2,000 bp.

1% Agarose gel, pre-stain, 100V, 20mins.

1. **End-prep:**

Materials: ~ 1 μg captured DNA in 50 μL

Consumables: NEBNext End Repair/ dA-tailing Module (E7546), 1.5 mL Eppendorf DNA LoBind tubes, 0.2 mL thin-walled PCR tube, AMPure XP beads, Absolute ethanol, Nuclease-free water, 10 mM Tris-HCl pH 8.0 buffer

Equipment: Thermal cycler, Microfuge, Vortex, Qubit Fluorometer, Magnetic Rack (1.5 mL)

1. Mix the following reagents in a 0.2 mL thin-walled PCR tube:

| Reagent | Volume |
| --- | --- |
| ~1 μg DNA | 50 μL |
| Ultra II End-prep reaction buffer | 7 μL |
| Ultra II End-prep enzyme mix | 3 μL |
| Total | **60 μL** |

1. Mix gently by flicking the tube, and spin down.
2. Incubate the reaction for 15 minutes at 20° C and 15 minutes at 65° C in a thermal cycler.
3. Transfer the sample to a 1.5 mL DNA LoBind Eppendorf tube.
4. Prepare AMPure XP beads for use; resuspend by vortexing.
5. Add 0.6x of resuspended AMPure XP beads to the end-prep reaction and mix gently by pipetting.
6. Incubate for 10 minutes at RT, agitating every few minutes.
7. Prepare 500 μL of fresh 80% ethanol in nuclease-free water.
8. Spin down the sample and pellet on a magnet until the supernatant is clear and colourless. Keep the tube on the magnet, and pipette off the supernatant into a clean 1.5 mL Eppendorf DNA LoBind tube (labeled as supernatant).
9. Keep on magnet, wash beads with 200 µL of freshly prepared 80% ethanol without disturbing the pellet.
10. Remove the 80% ethanol using a pipette and discard.
11. Repeat steps 9.10-9.11.
12. Spin down and place the tube back on the magnet. Pipette off any residual ethanol.
13. Allow to dry for ~2 minutes, but do not dry the pellet to the point of cracking.
14. Remove the tube from the magnetic rack and resuspend the pellet in 32 µL of Tris-HCl buffer.
15. Gently agitate the tube to ensure that beads are covered in the buffer and incubate for 10 minutes at RT, gently agitating the tube every few minutes to aid resuspension of the pellet.
16. Pellet the beads on a magnet until the eluate is clear and colourless.
17. Extract 31 µL of eluate into a clean 1.5 mL Eppendorf DNA LoBind tube.
18. Analyse 1 µL of the end-prepped DNA from 9.18 and supernatant from 9.9 for:

Qubit:

| **End-Prepped DNA** | µg/μL | total quantity (µg) |
| --- | --- | --- |
| sample |  |  |
| sample supernatant |  |  |

1. **Adapter Ligation:**

Materials: End-repaired DNA in 30 µL, Adapter Mix (AMX), Short fragment buffer (SFB), Elution Buffer (EB), Ligation Buffer (LNB).

Consumables: NEBNext Quick Ligation Module (E6056), 1.5 mL Eppendorf DNA LoBind tubes, AMPure XP beads, 10 mM Tris-HCl pH 8.0 buffer

Equipment: Microfuge, Vortex, Qubit Fluorometer, Magnetic Rack (1.5 mL), Ice Bucket with Ice

1. Thaw and prepare the kit reagents as follow:

| Contents* | On Ice | At RT | Remarks |
| --- | --- | --- | --- |
| Adaptor Mix | Y |  |  |
| T4 ligase from NEBNext Quick Ligation Module (E6056) | Y |  |  |
| Ligation Buffer (LNB) |  | Y | spin down and mix by pipetting then place on ice |
| Elution Buffer (EB) |  | Y | Mix by vortex, spin down then place on ice |
| S Fragment Buffer (SFB) |  | Y | Mix by vortex, spin down then place on ice |

******* *reagents should have no precipitants*

1. Prepare adaptor ligation mastermix in 1.5 mL Eppendorf DNA LoBind tube:

| Reagent | Volume |
| --- | --- |
| End-repaired DNA from the previous step | 30 μL |
| 10 mM Tris-HCl pH 8.0 | 30 μL |
| Adapter Mix (AMX) | 5 μL |
| Ligation Buffer (LNB) | 25 μL |
| NEBQuick T4 DNA ligase | 10 μL |
| Total | **100 μL** |

1. Mix gently by flicking the tube, and spin down.
2. Incubate the reaction for 10 minutes at RT.
3. Prepare the AMPure XP beads for use; resuspend by vortexing.
4. Add 0.6x of resuspended AMPure XP beads to adapt the ligation reaction and mix gently by pipetting.
5. Incubate for 10 minutes at RT, agitating every few minutes.
6. Spin down the sample and pellet on a magnet until the supernatant is clear and colourless. Keep the tube on the magnet, and pipette off the supernatant into a clean 1.5 mL Eppendorf DNA LoBind tube (labeled as supernatant).
7. Add 250 μL of the S Fragment buffer (SFB) to the beads.
8. Close the tube lid and resuspend the beads by flicking the tube.
9. Return the tube to the magnetic rack, allow beads to pellet and pipette off the supernatant.
10. Repeat 10.9-10.11.
11. Spin down and place the tube back on the magnet. Pipette off any residual buffer.
12. Allow to dry for 2 minutes.
13. Remove the tube from the magnetic rack and resuspend pellets in 14 µL of Elution Buffer (EB).
14. Incubate for 10 minutes at RT.
15. Pellet the beads on a magnet until the eluate is clear and colourless.
16. Extract 13 µL of eluate into a clean 1.5 mL Eppendorf DNA LoBind tube.
17. Quantify 1µl of eluted sample using Qubit Fluorometer:

Qubit:

| **Adapter-ligated DNA** | µg/µL | Total quantity (µg) | Amount in fmoles |
| --- | --- | --- | --- |
| sample |  |  |  |
| sample supernatant |  |  | / |

<https://worldwide.promega.com/resources/tools/biomath/>

1. Store the library on ice until loading into the flow cell.
2. **Priming and loading the SpotON flow cell:**

Materials: DNA library in 12 µL, Flush Tether (FLT), Flush Buffer (FLB), Sequencing Buffer (SQB) and Loading Beads (LB)

Consumables: SpotON Flow Cell

Equipment: MinION

1. Thaw the Sequencing Buffer (SQB), Loading Beads (LB), Flush Tether (FLT) and one tube of Flush Buffer (FLB) at room temperature and place the tubes on ice as soon as thawing is complete.
2. Mix the Sequencing Buffer (SQB) and Flush Buffer (FLB) tubes by vortexing, spin down and return to ice.
3. Spin down the Flush Tether (FLT) tube, mix by pipetting, and return to ice.
4. Flip back the MinION lid.
5. Slide the priming port cover clockwise to open the priming port.
6. Set a P1000 pipette to 200 µL and insert the tip into the priming port.
7. Turn the wheel until the dial shows 220-230 µL, or until you can see a small volume of buffer entering the pipette tip to remove any bubbles. ***Do not remove more than 20-30 µL of buffer.***
8. ***Visually check that there is a continuous buffer from the priming port across the sensor array.***
9. **Prepare the flow cell priming mix**: add 30 µL of Flush Tether (FLT) directly to the tube of Flush Buffer (FLB), and mix by pipetting up and down.
10. Load 800 µL of the priming mix into the flow cell via the priming port, avoiding the introduction of air bubbles.
11. Wait for 5 minutes.
12. Thoroughly mix the contents of the Loading beads (LB) tube by pipetting.
13. Prepare the library for loading as follows:

| Reagent | Volume |
| --- | --- |
| Sequencing Buffer (SQB) | 37.5 μL |
| Loading Beads (LB), mixed immediately before use | 25.5 μL |
| 100 fmol DNA Library | 12 μL |
| Total | **75.0 µL** |

1. Gently lift the SpotON sample port cover to make the SpotON sample port accessible.
2. Load 200 µL of the priming mix into the flow cell via the priming port (not the SpotON sample port), avoiding the introduction of air bubbles.
3. Mix the prepared library gently by pipetting up and down just prior to loading
4. Add 75 μL of sample to the flow cell via the SpotON sample port in a dropwise fashion. Ensure each drop flows into the port before adding the next.
5. Gently replace the SpotON sample port cover, making sure the bung enters the SpotON port, close the priming port and replace the MinION lid.
