# Supplementary material for: ECNano: A cost-effective workflow for target enrichment sequencing and accurate variant calling on 4800 clinically significant genes using a single MinION flowcell
Source: BMC Med Genomics. 2022 Mar 4;15:43. doi: 10.1186/s12920-022-01190-3 (PMC8895767; doi:10.1186/s12920-022-01190-3)
Supplement: Supplementary file 3 — Additional file 3: The comparison in depth of coverage (DoC) between medical exome sequencing using ECNano ONT protocol and NGS Illumina NextSeq 500. The NGS data was obtained from a published sequencing run by Pengelly et al., 2020. For a fair comparison, random downsampling of the NGS reads was performed so that both runs have approximately 10Gbp total throughput. (B) Example depth distribution comparison of target regions in three captured genes: (i.e. AGRN, BRCA and NF1) between ECNano ONT (upper tracks) and NGS (lower tracks) sequencing using HG001 standard DNA samples. [file 12920_2022_1190_MOESM3_ESM.docx]

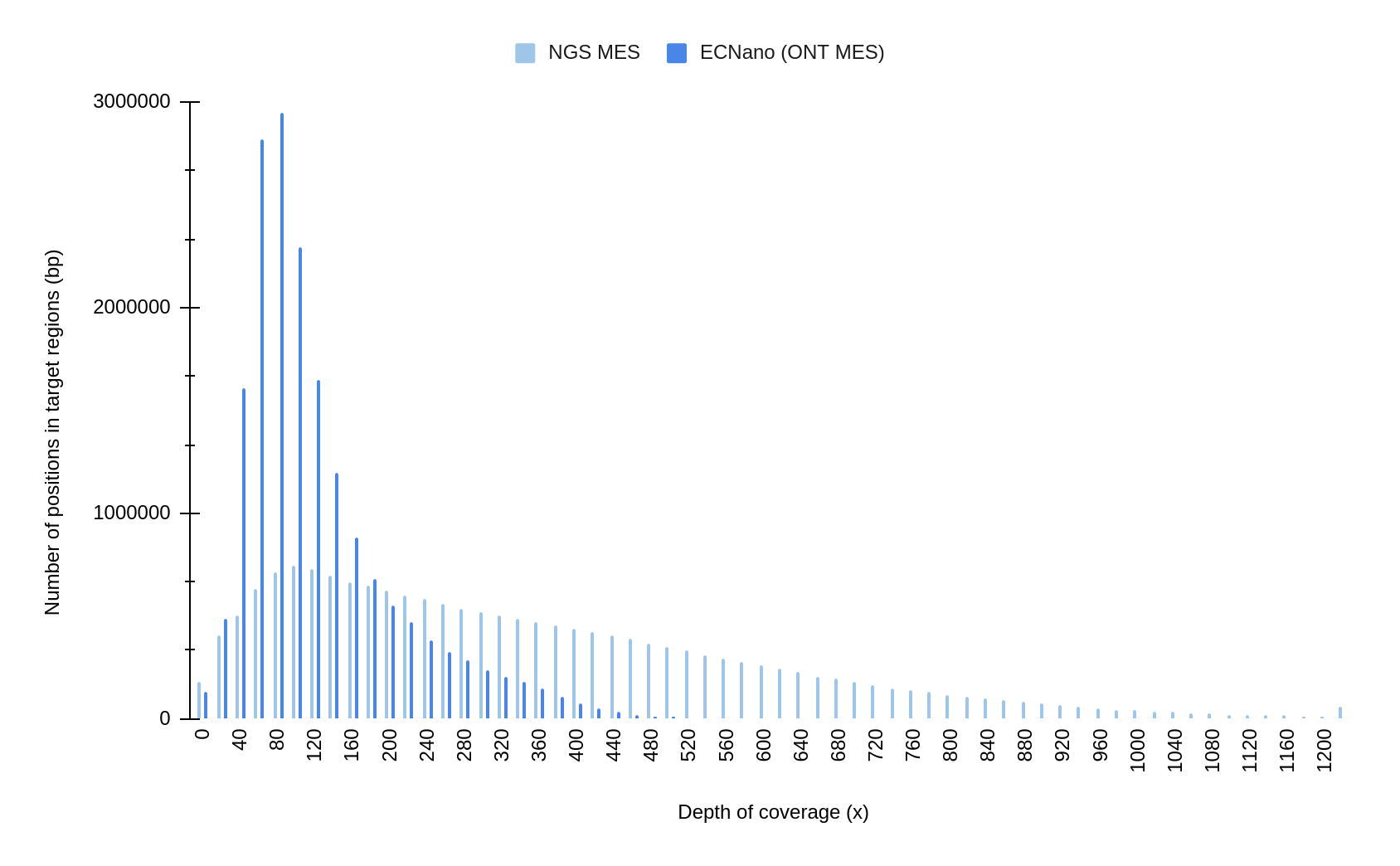


A.

(A) The comparison in depth of coverage (DoC) between medical exome sequencing using ECNano ONT protocol and NGS Illumina NextSeq 500. The NGS data was obtained from a published sequencing run by Pengelly et al., 2020. Both ONT and NGS used 10Gbp data for a fair comparison.


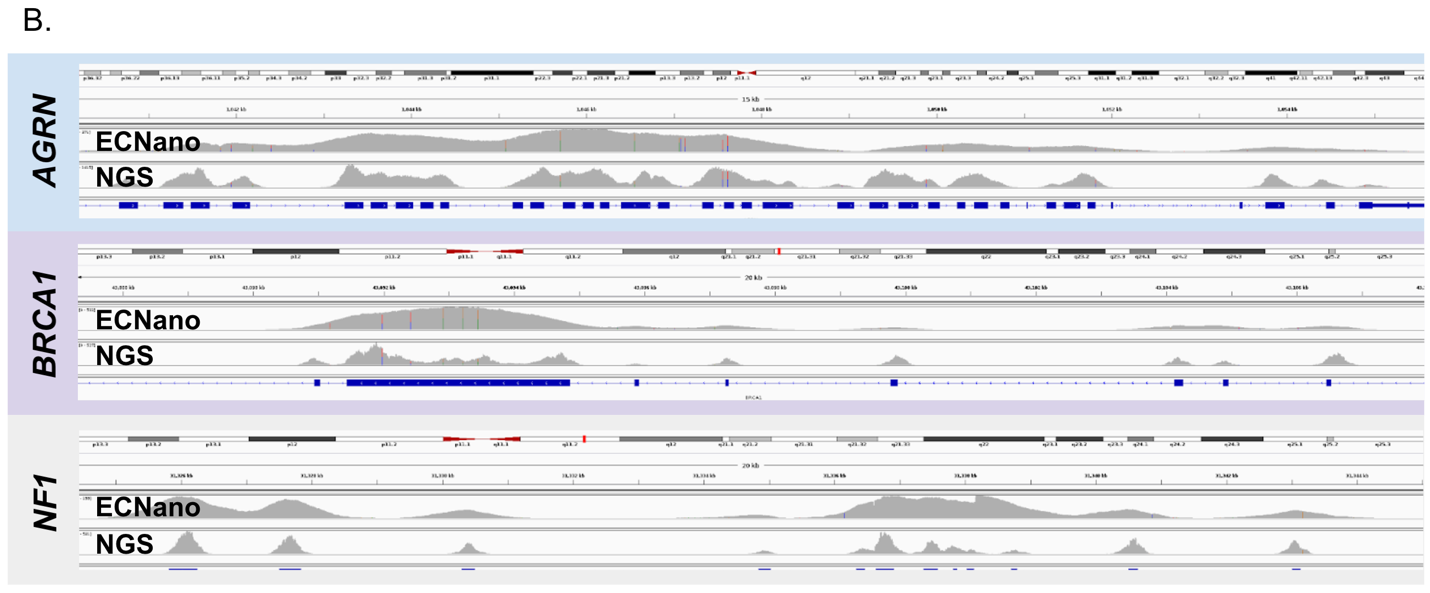


(B) Depth distribution comparison in the target regions in three genes: (including AGRN, BRCA and NF1) between ECNano ONT (upper tracks) and NGS (lower tracks) sequencing using the HG001 standard DNA sample.
